# Integrated Single-Cell and Spatial Transcriptomics Coupled with Machine Learning Uncovers *MORF4L1* as a Critical Epigenetic Mediator of Radiotherapy Resistance in Colorectal Cancer Liver Metastasis

**DOI:** 10.3390/biomedicines14020273

**Published:** 2026-01-26

**Authors:** Yuanyuan Zhang, Xiaoli Wang, Haitao Liu, Yan Xiang, Le Yu

**Affiliations:** 1 Department of Radiation Oncology, Jiangsu Cancer Hospital & Jiangsu Institute of Cancer Research & The Affiliated Cancer Hospital of Nanjing Medical University, Nanjing 210009, China; 2 Department of Pharmacy, Jiangsu Cancer Hospital & Jiangsu Institute of Cancer Research & The Affiliated Cancer Hospital of Nanjing Medical University, Nanjing 210009, China; 3 School of Life Sciences, Fudan University, Shanghai 200438, China; 4 Department of Nursing, Jiangsu Cancer Hospital & Jiangsu Institute of Cancer Research & The Affiliated Cancer Hospital of Nanjing Medical University, Nanjing 210009, China

**Keywords:** colorectal cancer liver metastasis, radiotherapy resistance, single-cell RNA sequencing, spatial transcriptomics, *MORF4L1*

## Abstract

**Background and Objective:** Colorectal cancer (CRC) liver metastasis (CRLM) represents a major clinical challenge, and acquired resistance to radiotherapy (RT) significantly limits therapeutic efficacy. A deep and comprehensive understanding of the cellular and molecular mechanisms driving RT resistance is urgently required to develop effective combination strategies. Here, we aimed to dissect the dynamic cellular landscape of the tumor microenvironment (TME) and identify key epigenetic regulators mediating radioresistance in CRLM by integrating cutting-edge single-cell and spatial omics technologies. **Methods and Results:** We performed integrated single-cell RNA sequencing (scRNA-seq) and spatial transcriptomics (ST) on matched pre- and post-radiotherapy tumor tissues collected from three distinct CRLM patients. Employing a robust machine-learning framework on the multi-omics data, we successfully identified *MORF4L1* (Mortality Factor 4 Like 1), an epigenetic reader, as a critical epigenetic mediator of acquired radioresistance. High-resolution scRNA-seq analysis of the tumor cell compartment revealed that the *MORF4L1*-high subpopulation exhibited significant enrichment in DNA damage repair (DDR) pathways, heightened activity of multiple pro-survival metabolic pathways, and robust signatures of immune evasion. Pseudotime trajectory analysis further confirmed that RT exposure drives tumor cells toward a highly resistant state, marked by a distinct increase in *MORF4L1* expression. Furthermore, cell–cell communication inference demonstrated a pronounced, systemic upregulation of various immunosuppressive signaling axes within the TME following RT. Crucially, high-resolution ST confirmed these molecular and cellular interactions in their native context, revealing a significant spatial co-localization of *MORF4L1*-expressing tumor foci with multiple immunosuppressive immune cell types, including regulatory T cells (Tregs) and tumor-associated macrophages (TAMs), thereby underscoring its role in TME-mediated resistance. **Conclusions:** Our comprehensive spatial and single-cell profiling establishes *MORF4L1* as a pivotal epigenetic regulator underlying acquired radioresistance in CRLM. These findings provide a compelling mechanistic rationale for combining radiotherapy with the targeted inhibition of *MORF4L1*, presenting a promising new therapeutic avenue to overcome treatment failure and improve patient outcomes in CRLM.

## 1. Introduction

Colorectal cancer liver metastasis (CRLM) remains the primary cause of mortality in patients with colorectal cancer [1]. Despite significant advancements in multimodal therapies, including surgery, chemotherapy, and radiotherapy (RT) [2], acquired radioresistance constitutes a critical bottleneck that severely limits clinical efficacy [3]. RT primarily exerts its cytotoxic effect by inducing massive DNA damage and generating abundant reactive oxygen species (ROS) in tumor cells [4,5]. However, surviving cancer cells often undergo complex transcriptional and epigenetic reprogramming to acquire potent anti-damage capabilities and adaptive features, ultimately leading to treatment failure and disease recurrence [6,7,8]. Therefore, there is an urgent need to dissect the dynamic and heterogeneous changes within the tumor microenvironment (TME) pre- and post-RT at single-cell and spatial resolution, and to identify the core molecular drivers mediating this resistant state.

Traditional genomic and transcriptomic analyses based on bulk tissue samples struggle to capture the inherent heterogeneity of rare cellular subpopulations within the TME and the intricate, cell-specific interactions between tumor and immune cells [9,10,11]. Single-cell RNA sequencing (scRNA-seq) offers unprecedented resolution, enabling the identification of unique transcriptional characteristics of malignant cells that survive after RT and the elucidation of potential molecular mechanisms of resistance [12,13]. Furthermore, Spatial Transcriptomics (ST) is crucial as it can anchor these molecular discoveries within their native tissue structure, providing essential context for understanding the spatial specificity of TME reprogramming and intercellular communication [14].

Given that radioresistance involves multi-layered molecular adaptations, including enhanced DNA damage repair (DDR) [15], metabolic reprogramming [16], and immune evasion [17], we hypothesized the existence of one or a few master epigenetic regulators responsible for coordinating these extensive resistance programs. Epigenetic regulatory factors, by rapidly and persistently adjusting gene expression patterns through changes in chromatin accessibility [18], are ideally positioned to orchestrate the tumor cell’s adaptive response to RT stress [7]. However, little is known regarding which specific epigenetic factors play a pivotal role in CRLM radioresistance and how they integrate intrinsic cellular adaptation with extrinsic TME immunosuppressive signaling.

In this study, we performed an integrated scRNA-seq and ST analysis on matched pre- and post-RT tissues collected from three CRLM patients. Employing a robust machine-learning framework on the single-cell transcriptomic data, we successfully identified *MORF4L1* (Mortality Factor 4 Like 1) as the core epigenetic regulator. Through subsequent pseudotime analysis, transcription factor activity prediction, and cell–cell communication modeling, we not only confirmed that *MORF4L1* drives the tumor cell’s intrinsic stress-survival and metabolic reprogramming programs but also, for the first time, revealed its mechanism for spatially exacerbating TME immunosuppression: by upregulating the IGF2 ligand, which activates the IGF2R/EGR1 signaling axis between tumor cells and myeloid cells. Our findings establish *MORF4L1* as a critical and druggable regulatory node in CRLM radioresistance, providing a compelling rationale for an innovative combined therapeutic strategy targeting *MORF4L1* alongside RT to overcome treatment obstacles.

## 2. Methods

### 2.1. Patient Samples and Data Acquisition

Matched tumor tissues were collected from three patients diagnosed with Colorectal Cancer Liver Metastasis (CRLM) at both pre-radiotherapy (RT) and post-RT timepoints, following ethical committee approval. All samples were rapidly processed for both single-cell RNA sequencing (scRNA-seq) and spatial transcriptomics (ST). Samples were dissociated and prepared following established protocols for the 10× Genomics platform.

### 2.2. Single-Cell RNA Sequencing Data Processing and Quality Control

Raw scRNA-seq data were processed using Cell Ranger (v6.0) to generate feature-barcode count matrices. Quality control (QC) was performed to exclude low-quality cells based on stringent criteria. Specifically, cells were retained only if they met the following three metrics: (1) detected gene count > 200 and <6000; (2) total UMI count > 1000; and (3) mitochondrial gene percentage < 15%. The resulting high-quality cell matrices were processed using the Seurat R package (v4.4.1) [19]. Data normalization was performed using the SCTransform method, followed by regression of total UMI counts and mitochondrial percentage to remove technical variance. Principal Component Analysis (PCA) was used for initial dimensionality reduction.

### 2.3. Cell Clustering and Annotation

The top 30 principal components were used to construct a Shared Nearest Neighbor (SNN) graph, which was then subjected to clustering via the Louvain algorithm (FindClusters function in Seurat). The Uniform Manifold Approximation and Projection (UMAP) method was applied for two-dimensional visualization. Cell populations were annotated based on the expression of canonical lineage-specific marker genes identified through differential expression analysis (FindAllMarkers function). The malignant cell compartment was extracted and re-clustered at a higher resolution for subsequent in-depth analysis.

### 2.4. Malignancy Validation and CNV Analysis

The malignant identity of tumor cell clusters was confirmed by inferring large-scale chromosomal copy number variations (CNVs). CNV scores were calculated using the inferCNV (v3.2.0) R package [20], using the combined non-malignant immune cell populations as a stable diploid reference. Clusters displaying significant and consistent levels of chromosomal gains and losses were definitively classified as malignant cells.

### 2.5. Transcriptional Reprogramming and Functional Analysis

Non-Negative Matrix Factorization (NMF): The normalized gene expression matrix of the purified malignant cells was subjected to NMF using the GeneNMF(v0.9.3) R package to resolve distinct gene expression programs (modules). The optimal number of modules (k = 10) was empirically determined by assessing the cophenetic correlation coefficient and residual sum of squares. Module scores were calculated to quantify module activity across pre- and post-RT states.

Gene Set Enrichment Analysis (GSEA): Functional enrichment of the genes contributing significantly to the upregulated modules (Module 4 and Module 9) was performed using the fgsea (v1.32.2) R package against the Molecular Signatures Database (MSigDB) HALLMARK gene sets to identify pathways related to resistance, metabolism, and immune response. To identify shared transcriptional programs driving resistance across patients, NMF was applied to the integrated gene expression matrix of purified malignant cells from all three patients. We selected k = 10 as the optimal number of metaprograms based on the point where the Cophenetic correlation coefficient began to decrease significantly, and the Residual Sum of Squares (RSS) curve showed an inflection point, ensuring the stability and biological interpretability of the consensus modules across the cohort.

Differential expression analysis was performed using the Wilcoxon Rank Sum test via the FindAllMarkers function in Seurat. To ensure statistical rigor, *p*-values were adjusted for multiple comparisons using the Bonferroni correction. Genes were considered significantly differentially expressed if they met the following criteria: an adjusted *p*-value (*p*-value_adj) < 0.05 and a log2 fold change (|log2FC|) > 0.25. For functional enrichment analyses (GSEA/GO), statistical significance was determined using a false discovery rate (FDR) < 0.05.

### 2.6. Pseudotime Trajectory Analysis

The temporal progression and developmental trajectory towards the radioresistant state were reconstructed using the Monocle (v2.24.0) R package on the malignant cell population [21]. Ordering genes were identified via differential expression analysis, and cells were subsequently ordered along the pseudotime axis using the DDRTree algorithm, with pre-RT cells defined as the initial state. The expression profile of *MORF4L1* was mapped onto the trajectory to assess its temporal correlation with resistance acquisition. Gene Ontology (GO) enrichment analysis was conducted on the top 100 genes positively correlated with pseudotime to functionally characterize the terminal-resistant state.

### 2.7. Machine-Learning-Based Feature Selection and Validation

To systematically identify candidate epigenetic regulators, three distinct feature selection algorithms were applied to the normalized malignant cell expression matrix: LASSO (Least Absolute Shrinkage and Selection Operator) regression, Boruta feature selection, and a Random Forest classifier. Genes robustly selected by the consensus of these three models were intersected with a comprehensive list of known human epigenetic regulatory genes. The predictive efficacy of the resulting candidates (e.g., *MORF4L1*) for distinguishing pre- and post-RT cells was evaluated using Receiver Operating Characteristic (ROC) curve analysis.

### 2.8. Spatial Transcriptomics (ST) Analysis

ST data were processed using the Space Ranger pipeline (v1.3) and analyzed with the Seurat (v5.2.1) R package. Spot-level normalization and scaling were performed. The spatial expression patterns of *MORF4L1* and specific immune cell markers were visualized, allowing for the quantification of co-localization between *MORF4L1*-high tumor regions and immune cell infiltration.

### 2.9. Cell–Cell Communication and Regulatory Network Analysis

Intercellular Communication: Ligand-receptor analysis, focusing on the communication axis between tumor cells (ligand source) and Myeloid cells (receptor target), was inferred using the NicheNet R package [22]. The analysis prioritized ligands secreted by tumor cells most likely to affect the transcriptional profile of Myeloid cells, resulting in the identification and prioritization of the IGF2-IGF2R axis. Downstream Target Prediction: For the prioritized IGF2-IGF2R axis, NicheNet was utilized to predict the most likely downstream target genes and regulatory transcription factors (e.g., EGR1) within the recipient Myeloid cells.

Transcription Factor (TF) Activity: TF activity in tumor cells was assessed using the Scenic R package to identify TFs (e.g., CUX1, ELF1, ETV4, YY1) exhibiting significantly altered activity post-RT [23].

### 2.10. Clinical Validation

The prognostic relevance of *MORF4L1* was externally validated using bulk RNA-seq and corresponding clinical data from The Cancer Genome Atlas (TCGA) Colorectal Cancer (CRC) cohort. Patients were dichotomized based on the median expression of *MORF4L1*, and overall survival (OS) was compared using Kaplan–Meier survival analysis.

### 2.11. Code Availability

The custom code used in this study is not publicly available but can be obtained from the corresponding author upon reasonable request.

## 3. Results

### 3.1. Single-Cell Landscape of the Tumor Microenvironment Pre- and Post-Radiotherapy

To delineate the dynamic changes in the tumor microenvironment (TME) induced by radiotherapy (RT), we performed single-cell RNA sequencing (scRNA-seq) on matched tumor tissues collected from three distinct colorectal cancer liver metastasis (CRLM) patients, sampling both pre- and post-RT timepoints.

**Figure 1:**
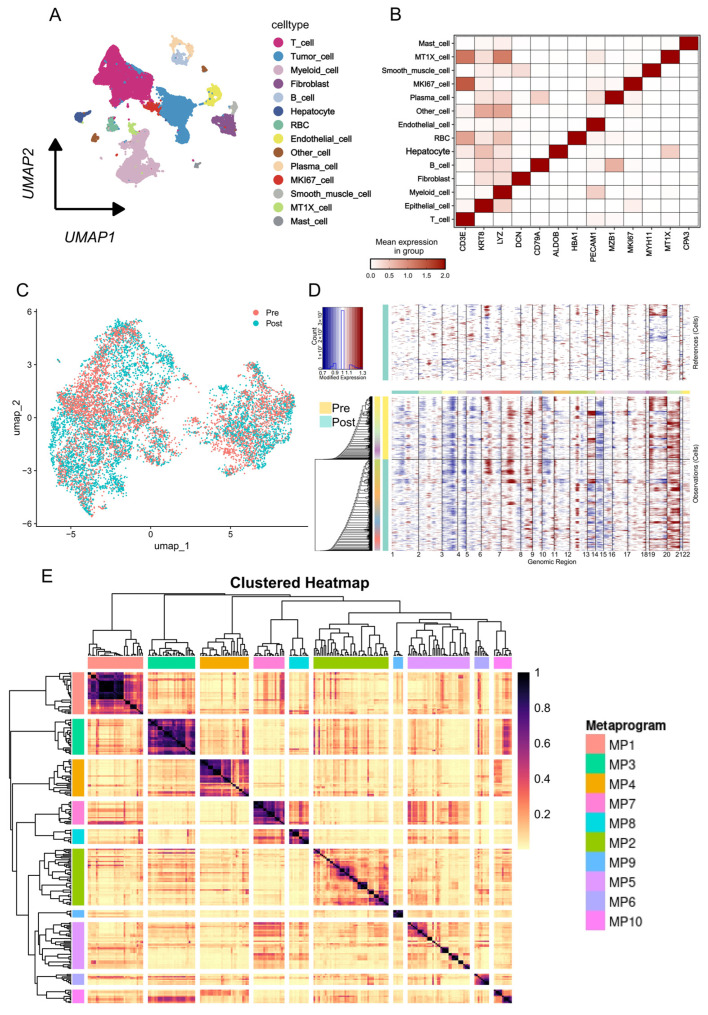
Single-Cell Landscape of the Tumor Microenvironment Pre- and Post-Radiotherapy: (**A**) Uniform Manifold Approximation and Projection (UMAP) visualization of 14 major cell clusters identified by single-cell RNA sequencing (scRNA-seq) of Colorectal Cancer Liver Metastasis (CRLM) tissues, colored by annotated cell type. (**B**) Heatmap showing the mean expression of canonical marker genes across the identified cell types, validating cluster identities. (**C**) UMAP visualization of tumor cells, colored by treatment group (Pre-RT vs. Post-RT), demonstrating the mixed nature of the samples. (**D**) Malignancy confirmation via copy number variation (CNV) analysis. Heatmap of inferred chromosomal aberrations across pre- and post-RT tumor cells. (**E**) Clustered heatmap showing the results of Non-Negative Matrix Factorization (NMF) applied to the malignant cell compartment, resolving tumor heterogeneity into 10 distinct gene expression metaprograms (MP1–MP10).

Cell clusters were rigorously annotated based on the expression of canonical marker genes, leading to the identification of 14 distinct cell populations (Figure 1A). These populations encompassed all major cellular components of the TME (Figure 1B), including Tumor cells, T cells, Myeloid cells, Fibroblasts, B cells, Plasma cells, Endothelial cells, and Mast cells. We also identified specific non-malignant populations, such as Hepatocytes and Red Blood Cells (RBCs), and functional subsets, including MKi67+ proliferative cells and MT1X+ stress-response cells, along with one minor other cell cluster.

We next performed a high-resolution re-clustering of the tumor cell compartment (Figure 1C). To confirm the malignant nature of these cells and validate their inclusion in subsequent analyses, we conducted a copy number variation (CNV) analysis, using the immune cell populations as a diploid reference. Significant levels of large-scale chromosomal aberrations were detected across both pre- and post-RT tumor cell clusters, unequivocally confirming their malignant phenotype (Figure 1D). This validated malignant population was then prioritized for subsequent mechanistic investigation into radioresistance.

### 3.2. Transcriptional and Functional Reprogramming in Post-Radiotherapy Tumor Cells

**Figure 2:**
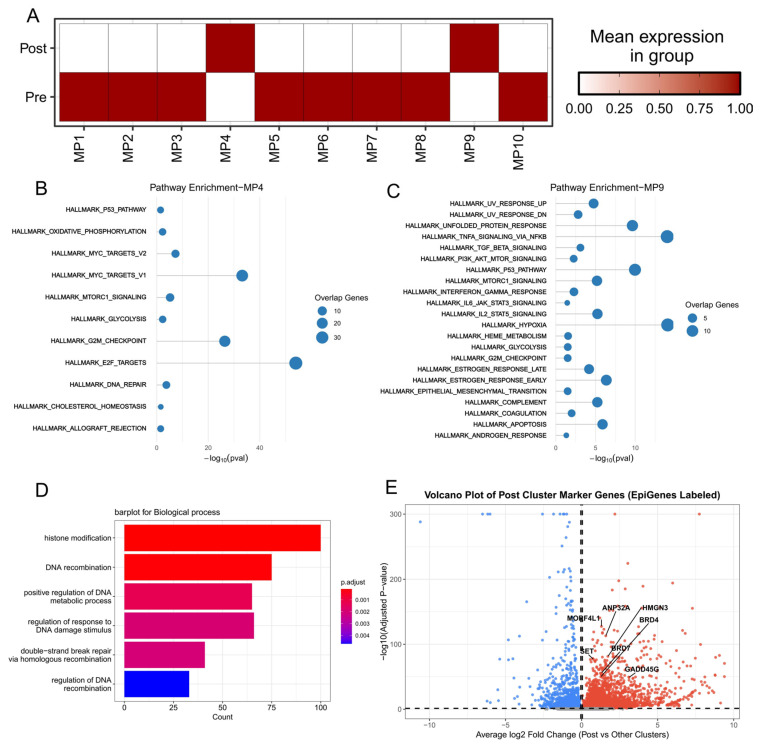
Transcriptional Reprogramming and Functional Adaptation in Post-Radiotherapy Tumor Cells: (**A**) Heatmap illustrating the mean module score activity of the 10 NMF metaprograms in pre-RT and post-RT malignant cells, highlighting the significant and consistent upregulation of Module 4 and Module 9 in post-RT cells. (**B**) Gene Set Enrichment Analysis (GSEA) bubble plot for Module 4, showing strong enrichment for pathways crucial for radioresistance. (**C**) GSEA bubble plot for Module 9, showing enrichment for immune evasion and stress pathways. (**D**) Barplot of Biological Process Gene Ontology (GO) terms enriched in differentially expressed genes (DEGs) between pre- and post-RT tumor cells, emphasizing chromatin and DNA modification processes. (**E**) Volcano plot highlighting differentially expressed epigenetic regulator genes in post-RT cells (Post Cluster) compared to other clusters, showing *MORF4L1* as a significantly upregulated candidate. In the plot, red represents genes with increased expression levels after radiotherapy, while blue represents those with decreased expression levels.

To investigate the underlying transcriptional adaptations of tumor cells following RT exposure, we applied Non-Negative Matrix Factorization (NMF) to the scRNA-seq data from the purified malignant cell compartment. This analysis resolved the tumor cell heterogeneity into 10 distinct gene expression modules (Figure 1E). Comparing the module expression profiles between pre- and post-RT samples, we observed a significant and consistent upregulation of Module 4 and Module 9 specifically in the post-treatment tumor cells (Figure 2A).

Functional enrichment analysis (GSEA) of the genes within these two upregulated modules provided critical insights into the acquired resistance mechanisms (Figure 2B,C).

Module 4 genes were predominantly associated with pathways crucial for cell survival, proliferation, and damage mitigation under stress. Key enrichments included: DNA Damage Response (DDR): DNA_REPAIR, regulation of response to DNA damage stimulus, and double-strand break repair via homologous recombination. The robust activation of these pathways highlights the tumor cells’ enhanced ability to repair RT-induced DNA lesions, a central mechanism of radioresistance. Metabolic Reprogramming: Strong enrichment of GLYCOLYSIS and OXIDATIVE_PHOSPHORYLATION, alongside CHOLESTEROL_HOMEOSTASIS, suggests a state of enhanced metabolic plasticity—a shift vital for providing energy and building blocks required for survival and accelerated repair.

Proliferation and Oncogenic Signaling: The activation of MTORC1_SIGNALING, MYC_TARGETS_V1/V2, E2F_TARGETS, and G2M_CHECKPOINT supports a globally aggressive, highly proliferative phenotype maintained by the surviving cells. The enrichment of P53_PATHWAY in this context suggests either an active p53-mediated response or, more likely, a mechanism to evade p53-induced apoptosis.

Module 9 was strongly enriched in pathways indicative of inflammation and immune evasion, consistent with TME-driven resistance: Pro-Survival and Immune Evasion: IL6_JAK_STAT3_SIGNALING is a potent signaling cascade known to promote cancer cell survival, anti-apoptosis, and the creation of an immunosuppressive environment.

T Cell Modulation and Stress: IL2_STAT5_SIGNALING and ALLOGRAFT_REJECTION suggest complex interactions with T cells, potentially indicating the recruitment or differentiation of immunosuppressive T cell subsets. Furthermore, the strong enrichment for HYPOXIA reflects the harsh microenvironmental stress caused by RT, which fuels metabolic shifts and resistance.

Differential gene expression analysis (DEGs) between pre- and post-RT tumor cells further confirmed these findings, with pathways related to chromatin and DNA modification being significantly enriched, including histone modification, DNA recombination, and regulation of the DNA metabolic process. To pinpoint the core regulatory mechanism mediating these widespread transcriptional changes, we interrogated the set of upregulated DEGs for known epigenetic regulators. This analysis revealed a panel of epigenetic-modifying genes that were substantially and uniquely upregulated in the post-RT tumor cells, collectively indicating a profound alteration in the malignant cell epigenome. These convergent results strongly suggest that RT alters the tumor’s epigenetic landscape, which subsequently fuels the enhanced survival, metabolic adaptation, and exacerbated immune suppression, ultimately leading to acquired radioresistance.

### 3.3. Machine Learning Identifies MORF4L1 as the Central Epigenetic Driver of Radioresistance

**Figure 3:**
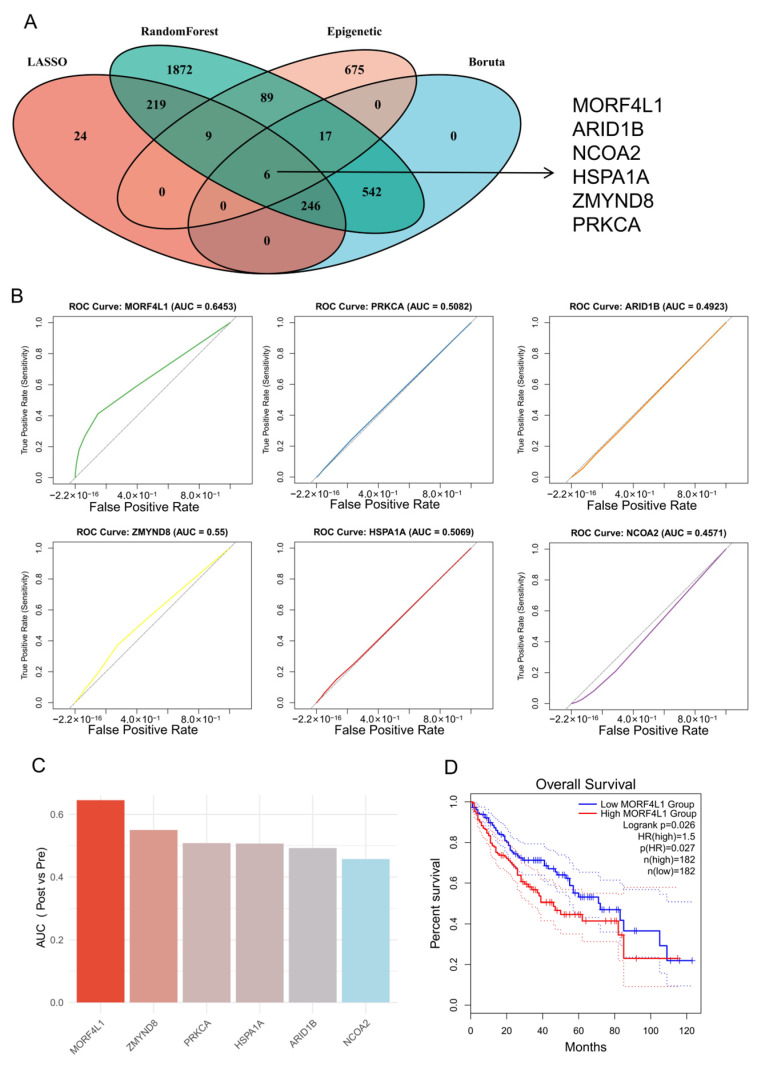
Machine Learning Identifies *MORF4L1* as the Central Epigenetic Driver: (**A**) Venn diagram illustrating the consensus selection of candidate epigenetic regulators by three distinct machine learning algorithms (LASSO regression, Boruta feature selection, and Random Forest classifier) applied to the malignant cell expression data. The intersection identifies the final six robust candidates. (**B**) Receiver Operating Characteristic (ROC) curves for the six candidate epigenetic regulators, demonstrating their predictive efficacy for classifying pre- vs. post-RT tumor cells. *MORF4L1* shows the highest Area Under the Curve (AUC). (**C**) Bar plot summarizing the AUC values for the six robust candidates, confirming *MORF4L1* has the highest predictive capacity for the radioresistant state. (**D**) Kaplan–Meier survival curve for The Cancer Genome Atlas (TCGA) Colorectal Cancer (CRC) cohort, stratified by high (above median) versus low (below median) *MORF4L1* expression, validating MORF4L1’s association with significantly poorer overall survival. Solid lines represent the survival probability, and the corresponding dashed lines indicate the 95% confidence intervals.

To systematically prioritize the key epigenetic regulators responsible for the observed transcriptional shift, we employed an integrated machine-learning approach. We utilized three distinct feature selection algorithms—LASSO regression, Boruta feature selection, and Random Forest classifier—on the pre- and post-RT scRNA-seq malignant cell dataset (Figure 3A). The consensus features identified by these robust models were then intersected with a comprehensive list of known epigenetic regulatory genes.

This rigorous filtering process yielded six robust candidate epigenetic regulators significantly associated with acquired radioresistance: *MORF4L1*, *ARID1B*, *NCOA2*, *HSPA1A*, *ZMYND8*, and *PRKCA*.

To further assess the predictive power and robustness of these candidates as single-cell biomarkers for the radioresistant state, we performed Receiver Operating Characteristic (ROC) curve analysis. *MORF4L1* exhibited the highest predictive capacity, with an Area Under the Curve (AUC) value exceeding 0.6 (AUC = 0.6453), whereas the other five genes showed AUC values below this threshold (Figure 3B,C). We explicitly evaluated the inter-patient variability for our key findings. *MORF4L1* Expression: We performed a patient-stratified analysis and observed that *MORF4L1* was significantly upregulated in the post-RT tumor cells of all three patients individually, showing no significant heterogeneity in this specific resistance trend. It is important to note that this moderate AUC value is physiologically relevant, as it reflects the inherent limitations of scRNA-seq data, specifically the challenges of transcriptional dropouts and the transient, low-level expression characteristic of many epigenetic regulators in single cells, thus making *MORF4L1* the most potent and reliable single-cell feature. Finally, we leveraged bulk RNA-seq data from The Cancer Genome Atlas (TCGA) CRC cohort to validate the clinical relevance of *MORF4L1*. Patients with high *MORF4L1* expression demonstrated significantly poorer overall survival and clinical outcomes compared to those with low expression (Figure 3D). Collectively, the multi-faceted analysis—from scRNA-seq feature selection and robustness testing to external clinical validation—converged to identify *MORF4L1* as the most critical epigenetic component mediating acquired resistance to radiotherapy in CRLM.

### 3.4. Pseudotime Trajectory Analysis Confirms MORF4L1 as a Hallmark of the Resistant State

**Figure 4:**
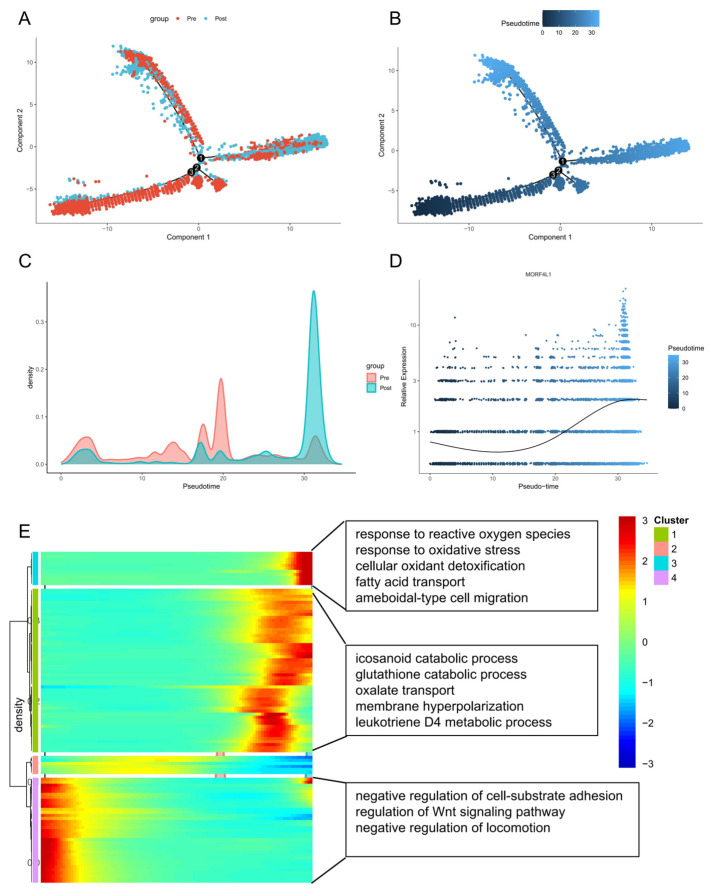
Pseudotime Trajectory Confirms *MORF4L1* as a Hallmark of the Resistant State (**A**) Pseudotime trajectory of malignant cells, colored by treatment group (Pre-RT: red, Post-RT: blue), showing the cells shifting towards the terminal state post-RT. The numbers (1, 2, 3) indicate the branch points of the differentiation trajectory. (**B**) The same pseudotime trajectory colored by pseudotime value (blue to light blue), representing the temporal progression toward radioresistance. (**C**) Density plot showing the distribution of pre-RT and post-RT tumor cells along the pseudotime axis, with post-RT cells highly concentrated at the terminal end (high pseudotime values). The black solid line represents the smoothed gene expression trend over pseudotime. (**D**) Scatter plot showing *MORF4L1* expression overlaid on the pseudotime axis, revealing a significant and monotonic increase in *MORF4L1* expression as cells progress towards the radioresistant state. (**E**) Heatmap of Gene Ontology (GO) enrichment analysis for the top 100 genes positively correlated with pseudotime, revealing functional characteristics of the terminal resistant state, including Antioxidant Defense, Cellular Oxidant Detoxification, and Metabolic Reprogramming.

To reconstruct the temporal and functional sequence of the malignant cells’ response to RT and validate the role of *MORF4L1*, we performed a pseudotime trajectory analysis on the re-clustered tumor cell population (Figure 4A). The resulting differentiation trajectory clearly separated the cells based on treatment status: the majority of pre-RT tumor cells occupied the early segments of the pseudotime axis, representing the initial, radiosensitive state, while post-RT tumor cells were overwhelmingly positioned at the terminal end, confirming the trajectory represents the biological progression towards acquired radioresistance (Figure 4B,C).

Crucially, integrating the expression of our lead candidate, *MORF4L1*, onto this trajectory showed a significant and monotonic increase along the pseudotime axis (Figure 4D). This direct correlation provides compelling evidence that the upregulation of *MORF4L1* is not merely an outcome but a central molecular event driving the transition to the stable radioresistant phenotype.

To characterize the underlying mechanisms driving this pseudotime progression, we performed Gene Ontology (GO) enrichment analysis on the top 100 genes whose expression was positively correlated with pseudotime (Figure 4E). The enriched pathways highlighted a state of profound cellular adaptation and stress resilience. Antioxidant Defense and Stress Survival: Strong enrichment was observed for response to reactive oxygen species, response to oxidative stress, and cellular oxidant detoxification. This demonstrates that cells at the terminal resistant state have dramatically enhanced their ability to neutralize RT-induced free radicals, a key survival strategy. Lipid and Energy Metabolism Reprogramming: Pathways related to lipid handling (fatty acid transport), eicosanoid signaling (icosanoid catabolic process, leukotriene D4 metabolic process), and generalized detoxification (glutathione catabolic process) were enriched. These metabolic shifts are essential for providing energy and building blocks required for accelerated repair and stress tolerance. Cellular Behavior and Signaling: The enrichment of ameboidal-type cell migration suggests an acquired, highly motile, and invasive phenotype, often associated with aggressive disease. Furthermore, terms like oxalate transport and membrane hyperpolarization point to altered ion homeostasis and signaling necessary for maintaining cell function under prolonged stress. Collectively, these pseudotime results validate that *MORF4L1* expression is intrinsically linked to the temporal acquisition of radioresistance, which is functionally characterized by enhanced antioxidant defenses and significant metabolic and migratory plasticity.

### 3.5. Spatial Validation and Inter-Cellular Communication Dynamics Post-Radiotherapy

**Figure 5:**
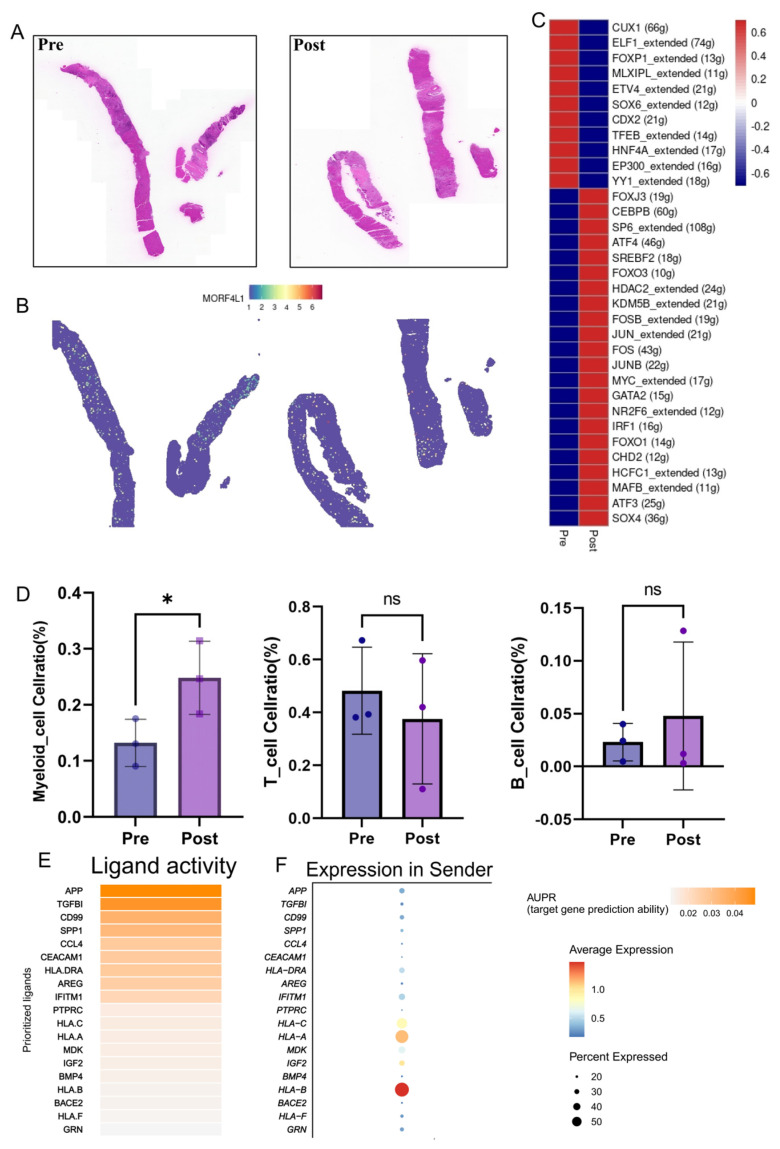
Spatial Validation and Inter-Cellular Communication Dynamics Post-Radiotherapy: (**A**) Hematoxylin and Eosin (H & E) staining of representative pre-RT and post-RT CRLM tissue sections. (**B**) Spatial transcriptomics (ST) mapping of *MORF4L1* expression, demonstrating its concentrated and higher expression in specific tumor regions post-RT. (**C**) Heatmap showing the inferred activity of key transcription factors (TFs) in pre- vs. post-RT tumor cells, highlighting increased activity of oncogenic TFs such as CUX1, ELF1, ETV4, and YY1 following RT. (**D**) Bar plots showing the proportions of Myeloid cells, T cells, and B cells. Statistical significance between groups was assessed using a paired two-tailed *t*-test. *, *p* < 0.05; ns, not significant. (**E**) Heatmap illustrating the activity of prioritized ligands secreted by tumor cells, highlighting significant upregulation of multiple immunosuppressive ligands, including IGF2. (**F**) Dot plot showing the average expression and percentage of cells expressing the prioritized ligands in sender tumor cells, confirming IGF2 upregulation.

To rigorously validate the pivotal role of *MORF4L1* in its native tissue context and assess its impact on the tumor microenvironment (TME) architecture, we utilized spatial transcriptomics (ST). The spatial expression mapping confirmed that *MORF4L1* expression was significantly higher and concentrated in specific tumor regions in post-RT samples compared to pre-RT tissues (Figure 5A,B). This spatial enrichment directly links *MORF4L1* to the morphologically confirmed radioresistant areas.

As an epigenetic regulator, *MORF4L1* likely exerts its effects by remodeling chromatin and driving transcriptional programs. To explore its functional consequence, we performed transcription factor (TF) activity analysis on post-RT tumor cells. The activity of several key oncogenic and survival-associated TFs, including CUX1, ELF1, ETV4, and YY1, was found to be substantially increased in the tumor cells following treatment (Figure 5C). The concurrent activation of these TFs, which are known to promote cell survival, invasion, and stemness, suggests that the *MORF4L1*-driven epigenetic reprogramming translates into a broad transcriptional activation of pro-survival and aggressive programs.

Furthermore, we investigated how RT alters the immune landscape. Examination of immune cell infiltration revealed a significant increase in the infiltration proportion of Myeloid cells post-RT, while the proportions of T cells and B cells showed no significant change (Figure 5D). The selective expansion of the myeloid compartment—which often adopts an immunosuppressive phenotype, acting as Myeloid-Derived Suppressor Cells (MDSCs) or tumor-associated macrophages (TAMs)—strongly implies an RT-induced shift toward an immunosuppressive TME.

**Figure 6:**
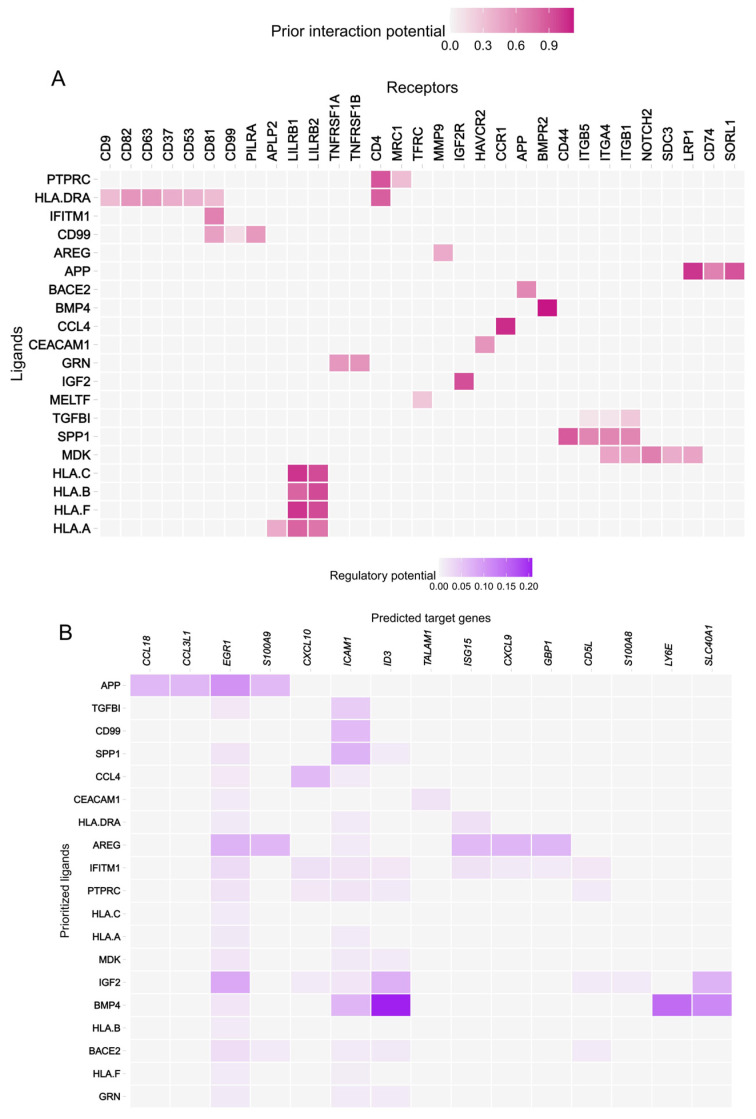
*MORF4L1*-Driven IGF2/EGR1 Axis Mediates Tumor-Myeloid Suppression: (**A**) NicheNet-predicted interaction potential between tumor-secreted ligands and myeloid cell receptors, identifying the IGF2-IGF2R axis as one of the highest-scoring interactions between the two cell types. (**B**) Regulatory potential heatmap showing the predicted downstream target genes and regulatory transcription factors in recipient Myeloid cells activated by the prioritized ligands (including IGF2), with EGR1 highlighted as a key transcription factor driving the immunosuppressive phenotype in myeloid cells.

To pinpoint the molecular dialogue fostering this immunosuppressive environment, we performed ligand-receptor interaction analysis focusing on the communication axis between tumor cells (ligand source) and myeloid cells (receptor target). The analysis revealed a robust, RT-induced upregulation of several potent immunosuppressive and pro-survival ligands secreted by tumor cells, including SPP1, TGFB1, CCL4, AREG, and IGF2 (Figure 5E). Specifically, we observed a pronounced increase in the expression of IGF2 alongside elevated expression of major histocompatibility complex class I molecules (HLA-A, HLA-B, HLA-C) in post-RT tumor cells, suggesting both immune evasion and paracrine signaling activation (Figure 5F). Predictive ligand-receptor binding analysis scored the IGF2-IGF2R axis as one of the highest predicted interactions between tumor cells and myeloid cells (Figure 6A).

The predicted downstream effector analysis of IGF2 signaling further highlighted the transcription factor EGR1 (Early Growth Response 1) as a high-scoring target (Figure 6B). EGR1 is a known critical transcription factor that drives the immunosuppressive polarization and differentiation of macrophages and other myeloid cells. These findings indicate a novel paracrine loop: *MORF4L1* upregulation in tumor cells enhances IGF2 secretion, which subsequently acts on myeloid cells, activating the EGR1-driven program to promote TME immunosuppression.

Collectively, the spatial and cell communication analyses confirm that *MORF4L1* upregulation is both spatially restricted to resistant tumor regions and functionally acts as a master regulator that actively reprograms the TME toward a highly immunosuppressive state, primarily through enhanced tumor-myeloid cell communication mediated by the IGF2/EGR1 axis.

## 4. Discussion

Acquired radioresistance represents a major hurdle in the clinical management of colorectal cancer liver metastasis (CRLM). Our study provides a comprehensive analysis of the dynamic changes within the CRLM tumor microenvironment (TME) pre- and post-radiotherapy (RT), leveraging cutting-edge integrated single-cell RNA sequencing (scRNA-seq) and spatial transcriptomics (ST).

Based on our integrated multi-omics analysis, our findings indicate that *MORF4L1* expression is dynamically upregulated in response to radiotherapy, rather than being driven by the selection of a pre-existing *MORF4L1*-high subpopulation. We support this conclusion with three lines of evidence: 1. Differential Expression: The volcano plot explicitly identifies *MORF4L1* as a top upregulated gene specifically in the Post-RT cluster compared to Pre-RT cells, indicating low baseline expression. 2. Pseudotime Trajectory: As shown in the trajectory analysis, *MORF4L1* expression exhibits a significant and monotonic increase along the pseudotime axis (from the initial pre-RT state to the terminal post-RT state), characterizing it as a marker of the acquired resistance process. 3. Spatial Confirmation: Our spatial transcriptomics data visually and quantitatively confirm that MORF4L1 protein/RNA levels are low in pre-RT tissues and become highly concentrated in surviving tumor regions post-RT. Most importantly, we successfully pinpointed *MORF4L1* (Mortality Factor 4 Like 1) as a critical epigenetic regulatory node, elucidating its role in driving both intrinsic tumor cell adaptation and extrinsic TME immunosuppression. This is the first report to define the core involvement of *MORF4L1* in acquired radioresistance at single-cell and spatial resolution.

Our data robustly established the molecular mechanism underlying tumor cell adaptation to RT stress. Non-Negative Matrix Factorization (NMF) analysis revealed that post-RT tumor cells exhibit significant upregulation in modules corresponding to enhanced DNA Damage Repair (DDR), metabolic reprogramming (Glycolysis and Oxidative Phosphorylation), and pro-survival signaling (e.g., mTORC1, MYC). The pseudotime trajectory analysis provided compelling temporal evidence, demonstrating that *MORF4L1* expression is positively and monotonically correlated with the progression of malignant cells toward the terminal radioresistant state (Figure 4A). This terminal state is functionally characterized by heightened antioxidant defense mechanisms (response to reactive oxygen species, cellular oxidant detoxification) and metabolic plasticity (fatty acid transport, glutathione catabolism). Regarding the temporal dynamics of resistance, our pseudotime analysis modeled a linear trajectory to capture the dominant evolutionary path driven by the strong selective pressure of radiotherapy. We acknowledge that tumor evolution is inherently complex and may involve branching pathways where distinct subclones develop resistance via alternative kinetics. However, the monotonic upregulation of *MORF4L1* observed along the primary pseudotime axis suggests a convergent adaptive response in the surviving population. Crucially, unlike resistance driven by fixed genetic mutations, the identification of *MORF4L1* as an epigenetic modulator implies that this radioresistant state is likely plastic and reversible. This theoretical reversibility suggests that the post-treatment phenotype might not be permanent and could potentially be reset or sensitized through targeted epigenetic intervention or withdrawal of the therapeutic stress. As a component of the NuA4/TIP60 acetyltransferase complex, *MORF4L1* is perfectly positioned to rapidly and persistently alter chromatin accessibility, thereby coordinating the sustained activation of these widespread survival programs. It is important to contextualize *MORF4L1* within the broader landscape of epigenetic radioresistance. Previous studies have implicated various regulators, including Histone Deacetylases (HDACs) and the SWI/SNF (BAF) chromatin remodeling complex, in the DNA damage response. Notably, our machine-learning consensus analysis also identified ARID1B, a core subunit of the BAF complex, as a robust candidate, validating our pipeline’s ability to capture established resistance mechanisms. However, *MORF4L1* emerged as the superior predictor with the highest AUC. While our current data establish a robust correlation, the precise mechanism by which *MORF4L1* drives IGF2 upregulation likely involves its canonical function as a component of the NuA4/TIP60 histone acetyltransferase complex [24]. As an epigenetic reader, MORF4L1 (via its chromo-barrel domain) recognizes specific histone modifications (e.g., H3K36me3) and recruits the NuA4/TIP60 complex to target gene loci. We postulate that in radioresistant cells, *MORF4L1* recruits this complex to the IGF2 promoter or enhancer regions. The subsequent acetylation of histone H4 and H2A by the TIP60 catalytic subunit would result in chromatin relaxation (open chromatin), thereby increasing the accessibility of the IGF2 locus to transcriptional machinery and upstream drivers identified in our network analysis. Furthermore, the concurrent increase in the activity of key oncogenic transcription factors—including CUX1, ELF1, ETV4, and YY1—in *MORF4L1*-high tumor cells suggests that the *MORF4L1*-driven epigenetic remodeling translates into a broad transcriptional activation of programs related to tumor aggressiveness, stemness, and invasion.

A critical insight from our study is the demonstration of how *MORF4L1* extends its influence beyond the tumor cell itself to reshape the TME. We observed a significant shift towards an immunosuppressive landscape post-RT, marked by a selective and pronounced increase in the infiltration of the Myeloid cell compartment, which typically adopts immunosuppressive phenotypes such as Myeloid-Derived Suppressor Cells (MDSCs) or Tumor-Associated Macrophages (TAMs). Cell–cell communication analysis, focused on the tumor-myeloid axis, identified a significant RT-induced upregulation of multiple immunosuppressive ligands (e.g., SPP1, TGFB1) secreted by tumor cells, most notably IGF2. Predictive binding analysis prioritized the IGF2-IGF2R interaction, suggesting a crucial paracrine loop. Downstream analysis further highlighted the transcription factor EGR1 (Early Growth Response 1), a known driver of immunosuppressive macrophage polarization, as a high-scoring target of IGF2 signaling. This defines a novel molecular cascade in which *MORF4L1* upregulation in tumor cells leads to enhanced IGF2 secretion, subsequently triggering IGF2R signaling activation in myeloid cells, and ultimately resulting in EGR1-driven immune suppression. The spatial transcriptomics data provide essential context, confirming that *MORF4L1* expression is concentrated in post-RT regions that spatially co-localize with increased immune cell infiltration, thus validating its role as a regional orchestrator of the immunosuppressive TME.

It is also crucial to contextualize our findings within the molecular heterogeneity of colorectal cancer. Our cohort consisted exclusively of patients with Microsatellite-Stable (MSS) CRLM, representing the vast majority (~95%) of metastatic cases. MSS tumors are typically characterized by a ‘cold’ immune microenvironment, distinct from the hyper-mutated and immune-infiltrated landscape of Microsatellite Instability-High (MSI-H) tumors. Therefore, the *MORF4L1*-IGF2-EGR1 immunosuppressive axis we identified may be specifically relevant to the MSS phenotype, potentially serving as a mechanism that reinforces the inherent immune exclusion in these tumors following radiotherapy. We acknowledge that our current data cannot determine whether this mechanism holds true across other molecular subtypes, such as MSI-H cancers or specific Consensus Molecular Subtypes (CMS), which possess fundamentally different baseline epigenetic and immunological states. Future studies stratified by molecular subtypes are warranted to define the boundaries of this mechanism’s applicability.

To address the challenge of generalizability given the heterogeneity of CRLM, future validation studies must be rigorously designed with adequate statistical power. Based on the robust effect sizes of *MORF4L1* expression differences observed in our single-cell analysis and the survival stratification in the TCGA cohort, future independent cohorts—specifically those consisting of MSS CRLM patients—should be sized to ensure sufficient power to detect these clinically relevant differences. Furthermore, for functional validation in preclinical models, sample sizes for in vivo experiments will be determined via power analysis to reliably assess the therapeutic synergy of *MORF4L1* inhibition combined with radiotherapy. This statistical rigor will be essential to confirm that the MORF4L1-IGF2-EGR1 axis is a prevalent and generalizable mechanism of resistance. Rationale for IGF2 as the Primary Target: As illustrated in our quantitative analysis of ligand expression (Figure 5F), IGF2 was identified as the dominant ligand in the post-RT tumor compartment. Its expression magnitude significantly eclipsed that of other potential candidates, including SPP1 and TGFB1 (ranking second only to the ubiquitous HLA class I molecules). Based on this data, we posit that IGF2 is the primary, non-redundant driver of the observed immunosuppression in our cohort, making it the most logical first-line therapeutic target.

In our study, the “Post-RT” samples were obtained at the end of the complete radiotherapy treatment course, after patients had undergone multiple fractions of radiation. Stable Epigenetic Change: Because these samples were not collected immediately after the first acute dose (which might capture transient early adaptation), the significant upregulation of *MORF4L1* observed in our data represents the cumulative outcome of the treatment. It indicates a stable, consolidated epigenetic state that was maintained or selected for throughout the weeks of therapy.

A primary limitation of our study is the relatively restricted cohort size (*n* = 3 patients), a constraint often inherent to high-resolution, biopsy-based scRNA-seq and spatial transcriptomics studies. While the longitudinal, paired design (pre- and post-RT from the same patients) significantly reduces the confounding effects of inter-patient variability, the small sample size may not fully capture the extensive inter-tumor heterogeneity characteristic of CRLM. Specifically, colorectal cancer encompasses diverse Consensus Molecular Subtypes (CMS) and mutational landscapes (e.g., KRAS, BRAF, or TP53 status), which could influence the baseline epigenetic state and the specific trajectory of radiotherapy resistance. Consequently, it remains to be determined whether the *MORF4L1*-driven resistance mechanism is a universal feature across all CRLM subtypes or specific to a particular molecular subclass. Although we validated the prognostic value of *MORF4L1* in the larger TCGA cohort, future multi-centric studies with larger sample sizes are essential to verify the prevalence of the MORF4L1-IGF2-EGR1 axis and to assess its generalizability across diverse patient demographics and clinical backgrounds. Furthermore, while our computational models strongly support this axis, we wish to be transparent that we do not currently have preliminary in vitro data regarding *MORF4L1* knockdown included in this manuscript. Our study was strictly designed as a clinical discovery effort, prioritizing the high-resolution profiling of rare, matched pre- and post-RT patient tissues using scRNA-seq and Spatial Transcriptomics. We believe the strength of our work lies in identifying this novel target within the complex, native human tumor microenvironment—a context that in vitro models often fail to fully recapitulate.

### Future Directions

To strictly validate the proposed mechanism, our future research will focus on a rigorous functional verification plan. First, at the cellular level, we will utilize MSS-subtype colorectal cancer cell lines to perform loss-of-function experiments, assessing the impact of *MORF4L1* knockdown on radiosensitivity via clonogenic survival assays and DNA damage repair markers such as gamma-H2AX. Second, we plan to verify the intercellular communication axis using tumor-macrophage co-culture systems, specifically focusing on the secretion of IGF2 and the downstream activation of EGR1 in myeloid cells. Finally, we intend to employ in vivo mouse models to evaluate whether targeting *MORF4L1* can synergize with radiotherapy to remodel the immunosuppressive tumor microenvironment and improve therapeutic outcomes.

In summary, our integrated multi-omics study establishes *MORF4L1* as a central epigenetic regulator that orchestrates acquired radioresistance in CRLM by concurrently promoting intrinsic survival mechanisms within tumor cells and fostering an immunosuppressive TME via the IGF2/IGF2R signaling axis. The validation of *MORF4L1*’s poor prognostic value in bulk TCGA data further strengthens its clinical relevance. These findings provide a robust mechanistic foundation for an innovative therapeutic strategy. Targeted inhibition of *MORF4L1* or its downstream signaling component, such as the IGF2/IGF2R axis, holds immense promise as a rational combination therapy with radiotherapy to effectively reverse both cellular resistance and TME-mediated immune evasion in CRLM patients.

## Data Availability

The datasets generated and analyzed during the current study are not publicly available due to patient privacy and institutional regulations, but are available from the corresponding authors on reasonable request.
